# Acetylation of *Amaranthus viridis* starch: Modeling and process parameters optimization

**DOI:** 10.1002/fsn3.677

**Published:** 2018-05-21

**Authors:** Temitope Omolayo Fasuan, Charles Taiwo Akanbi, Eriola Betiku

**Affiliations:** ^1^ Department of Food Science and Technology Obafemi Awolowo University Ile Ife Osun State Nigeria; ^2^ Department of Chemical Engineering Obafemi Awolowo University Ile Ife Osun State Nigeria

**Keywords:** acetic anhydride, acetylated starch, *Amaranthus viridis*, artificial neural network, response surface methodology

## Abstract

The optimum reaction conditions for the derivation of acetylated (esterified) starch using response surface methodology (RSM) and artificial neural network (ANN) were studied. All the independent variables (starch solids, acetic anhydride concentration, and reaction time) were of significant (*p *< .05) importance in achieving esterified starch of *Amaranthus viridis*. Optimum conditions of 152.46 g of starch, 11 ml of acetic anhydride and time of 2.92 min with corresponding acetyl content and degree of substitution (DS) of 1.74% and 0.06, respectively, were established for ANN. The RSM gave optimum conditions of 149.57 g (starch), 10.38 ml (acetic anhydride) and 3 min (time) with corresponding acetyl content and DS of 1.61% and 0.06, respectively. The order of priority of the process variables was established as acetic anhydride (42.59%), starch solids (33.90%), and reaction time (23.51%). The results provided useful information on development of economic and efficient acetylation process for modification of *A*.* viridis* starch.

## 1 INTRODUCTION

Genus *Amaranthus* contains over 60 species but only a few are cultivated, and many are considered weeds (Marin, Narcisa, & Popa, 2008). Amaranth is majorly cultivated for leaf and grains in many temperate and tropical regions. Amaranth is well known around the world and has become established for food use (both grain and leaves) in places like Africa, Central America, Southeast Asia, South America, and North America. The *Amaranthus* genotype species are cultivated as “pseudo cereals” due to their high content of carbohydrates, proteins and fats, comparable or even superior to cereals (Rusu, Marin, Moraru, Pop, & Cacovean, 2009; Toader & Roman, 2009). A seed of grain amaranth on average contains 13.1–21.0% crude protein; 5.6–10.9% crude fat; and 50–69% starch (Grobelnik‐Mlakar, Turinek, Jakop, Bavec, & Bavec, 2009). The average yield per hectare of *amaranthu*s in Nigeria is low (7.60 t/ha) relative to values reported from United States of America (77.27 t/ha) and the world average (14.27 t/ha) (FAO, 2007). *Amaranthus viridis* is an underutilized grain with little or no industrial application at present. However, its starch could have industrial applications as cheap alternative source of starch for food industry. *Amaranthus viridis* is easily cultivated with low labor cost and high grain yield compared to some other sources of starch such as cassava, corn, and breadfruit. Corn, cassava, and potato, which are major sources of starch for food industry, have some other domestic and industrial uses, which posed high demand on them. Moreover, the uses of starch in the food industry are becoming enormous at present. Therefore, there is need for alternative sources of starch from crops of lesser domestic and industrial demand.

Starch is an important food ingredient in the food industry. It is reported that about 53% of starch total production is used in the food sector (sweets – 18%, soft drinks – 11%, other foods – 24%; nonfood sector (total share of 46%); 28% is used for production of paper, cardboard and corrugated board, 13% is used for fermentation, and others 5% (Gérard, Colonna, Buléon, & Planchot, 2001; Hejazi et al., 2008). The uses of starch in food industry include frozen foods, dairy products, soups, sauces, canned foods, beverages, condiments, confectionery and gum, meat products, jams and jellies, syrups and sweeteners, and baking products. However, the limitations of native starch include low shear stress resistance, high retrogradation and syneresis, and poor solubility in common organic solvents (Kavlani, Sharma, & Singh, 2012). Therefore, the functionalities of starch in food industries can be enhanced through modifications. One of such modification techniques is acetylation (esterification). Esterification (often called acetylation) is a chemical modification mechanism, in which the hydroxyl groups are replaced with acetyl groups thereby leading to steric obstacles and a subsequent decrease in the gelatinization temperature. The acetylation reduces retrogradation and improves the stability at cooling and freezing points (Kavlani et al., 2012).

The starch properties of the seeds of some amaranth cultivars have been characterized (Baker & Rayas‐Duarte, 1998; Hoover, Sinnott, & Perera, 1998; Radosavljevic, Jane, & Johnson, 1998; Marcone, 2001; Choi, Kim & Shin, 2004). However, there is a dearth of information on modification of *A*. *viridis* starch, using the esterification (acetylation) technique. It is worthy of mentioning that the Design of Experiments (DOE) approach was not used in most of these reports on Amaranthus starch.

Modeling and optimization of processes involved in the food processing industry can be used to improve the yield of the target products. Rather than the typical one‐factor‐at‐a‐time method of optimization, which does not describe the complete effects of the variables in the process and does not consider the interactions between the variables, Response Surface Methodology (RSM), which defines the effect of the independent variables, alone or in combination in a process, is nowadays being applied in modeling and optimization studies (Bas & Boyaci, 2007; Betiku & Taiwo, 2015). This tool has been widely used in many areas of food research, such as production of dairy tofu (Chen, Chen, & Lin, 2005), ethanol production (Betiku & Taiwo, 2015), citric acid production (Betiku & Adesina, 2013; Dhillon, Brar, Verma, & Tyagi, 2011), lactic acid production (Naveena, Altaf, Bhadraya, Madhavenda, & Reddy, 2005) and oxalic acid production (Emeko, Olugbogi, & Betiku, 2015). Artificial Neural Network (ANN), which is a computational method that can mimics the neurological processing capability of the human brain, has also been applied to modeling of many food processing studies. These studies include gluconic acid (Osunkanmibi, Olowlabi & Betiku, 2015), ethanol (Betiku & Taiwo, 2015) and oxalic acid (Emeko et al., 2015) production processes as well as in enzymatic reaction catalyzed by amyloglucosidase (Bas & Boyaci, 2007). Many of these studies have demonstrated consistently that the predictive capability of ANN is stronger than RSM (Bas & Boyaci, 2007; Betiku & Taiwo, 2015; Emeko et al., 2015).

Therefore, this work aims to investigate the acetylation of *A*.* viridis* starch with the view to enhancing the utilization of the starch in food industry. The acetylation process was modeled, using both RSM and ANN. The vital variables of the acetylation process investigated include starch solids, acetic anhydride concentration, and the reaction time. The variables were optimized, using RSM and ANN coupled with genetic algorithm.

## 2 MATERIALS AND METHODS

### 2.1 Materials

*Amaranthus viridis* grains were cultivated by National Horticultural Research Institute (NIHORT), Kano, Nigeria and matured grains were collected after 14 weeks (April to July, 2015). The grains were wet cleaned and dried in a hot air oven (SM9053, Uniscope, UK) at 50°C for 8 hr. All chemicals were of analytical grades and obtained from Fisher Scientific (Oakville, ON, Canada) and Sigma Chemicals (St. Louis, MO, USA).

### 2.2 Isolation of starch from amaranth grains

**Figure 1:**
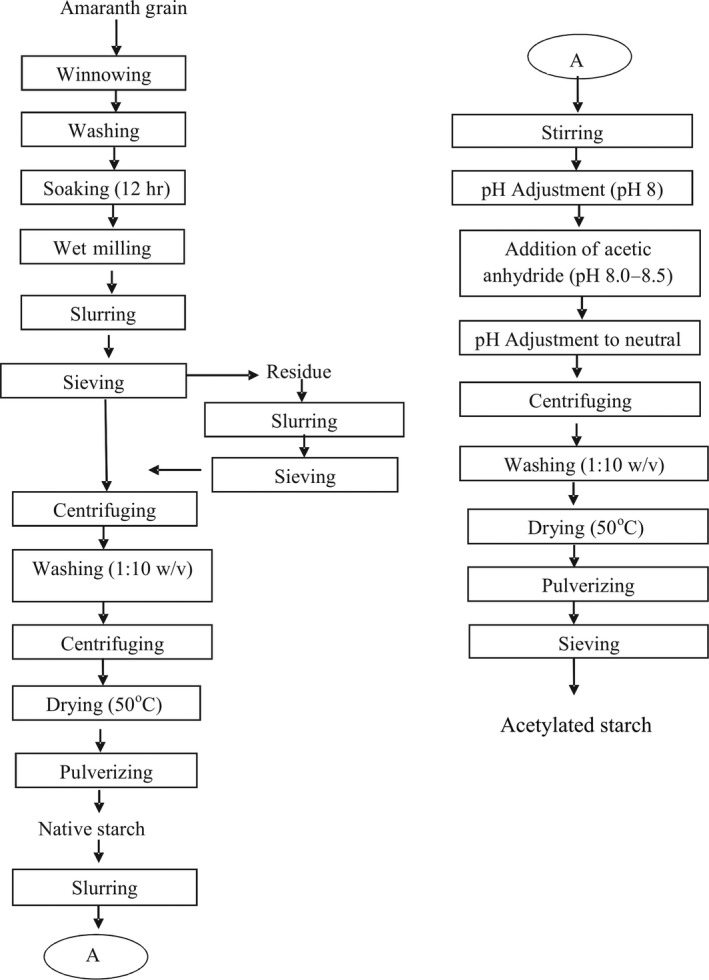
Flow chart for extraction and acetylation of *Amaranthus viridis* starch

Starch was extracted as described by Kong, Bao, and Corke (2009) with some modifications. The amaranth grains were soaked in distilled water (1:5, w/v) maintained at 28 ± 2°C for 12 hr. The seeds were rinsed, drained, and wet milled in attrition mill (Double Win, FS450, China). The resultant slurry was filtered through 149 μm mesh sieve. The filtrate was then centrifuged at 4552 × g, using a centrifuge (0502‐1, Hospibrand, USA) for 20 min. The starch layer was redispersed in distilled water (1:5 w/v), and centrifuged as earlier described, and this procedure was carried out in duplicate. The isolated starch was dried in an air oven (Uniscope, SM9053) at 50°C for 36 hr and ground, using a hammer mill to pass through a sieve with mesh sieve 212 μm. The flow chart for the process is as shown in Figure 1.

### 2.3 Experimental design and RSM modeling

The orthogonal central composite design (CCD) of RSM was used for this work. The three independent variables considered for the modeling include starch solid (50–150 g), acetic anhydride (5–15 ml) and time (3–13 min). The number of experimental conditions (Table 1) used for this work was generated using Equation (1), distance of the star points from the center point (2); real values of the center and star points were obtained, using Equations (3) and (4), respectively. The order of experimentation was completely randomized to avoid systematic errors.

**Table 1:** Acetyl content and degree of substitution of acetylated starch

Expt. No.	Run order	Starch solid (g)	Acetic anhydride (ml)	Time (min)	%Acetyl	Degree of substitution
1	3	−1 (50)	−1 (5)	−1 (3)	0.34 ± 0.01	0.01 ± 0.00
2	6	1 (150)	−1 (5)	−1 (3)	1.15 ± 0.41	0.05 ± 0.01
3	2	−1 (50)	1 (15)	−1 (3)	2.64 ± 0.37	0.10 ± 0.01
4	15	1 (150)	1 (15)	−1 (3)	1.53 ± 0.11	0.06 ± 0.00
5	1	−1 (50)	−1 (5)	1 (13)	0.97 ± 0.02	0.04 ± 0.00
6	9	1 (150)	−1 (5)	1 (13)	1.43 ± 0.02	0.05 ± 0.01
7	13	−1 (50)	1 (15)	1 (13)	2.09 ± 0.07	0.08 ± 0.01
8	20	1 (150)	1 (15)	1 (13)	0.57 ± 0.01	0.03 ± 0.00
9	5	−1.68 (15.9)	0 (10)	0 (8)	1.52 ± 0.01	0.06 ± 0.01
10	10	1.68 (184.1)	0 (10)	0 (8)	0.96 ± 0.02	0.04 ± 0.00
11	8	0 (100)	−1.68 (1.59)	0 (8)	0.68 ± 0.02	0.03 ± 0.00
12	16	0 (100)	1.68 (18.41)	0 (8)	1.99 ± 0.11	0.08 ± 0.01
13	11	0 (100)	0 (10)	−1.68 (0.41)	2.05 ± 0.14	0.08 ± 0.01
14	18	0 (100)	0 (10)	1.68 (16.41)	1.73 ± 0.09	0.07 ± 0.01
15	4	0 (100)	0 (10)	0 (8)	2.12 ± 0.04	0.09 ± 0.02
16	17	0 (100)	0 (10)	0 (8)	2.12 ± 0.12	0.09 ± 0.02
17	19	0 (100)	0 (10)	0 (8)	2.20 ± 0.08	0.09 ± 0.01
18	7	0 (100)	0 (10)	0 (8)	2.00 ± 0.02	0.09 ± 0.01
19	14	0 (100)	0 (10)	0 (8)	2.10 ± 0.01	0.08 ± 0.01
20	12	0 (100)	0 (10)	0 (8)	2.20 ± 0.02	0.09 ± 0.01

Values reported are means ± *SD* of triplicate determinations.

(1) $$n=2^{k}+2k+n_{0},$$

(2) $$\pm \propto =2^{k/4},$$

(3) $$\text{Centre point}=(X_{\mathrm{high}}{\;}_{\mathrm{level}}+X_{\mathrm{low}}{\;}_{\mathrm{level}})/2,$$

(4) $$\text{Star point}=X_{\mathrm{mean}}\pm \propto \left(\frac{X_{\mathrm{range}}}{2}\right),$$

where, *k* is the number of independent variables, *n*_o_ is the number of centre point and 2*k* is the number of star point, *X*_high level_ is value of independent variable at high level, *X*_low level_ is value of independent variable at low level, *X*_mean_ is mean of values of independent variable at low level and high level, *X*_range,_ is difference between values of independent variable at high level and low level.

The following statistical indicators were employed: coefficient of determination (*R* ^2^), adjusted (Adj. *R* ^2^), probability value at 95% confidence interval, predicted *R* ^2^, coefficient of variation, lack‐of‐fit, and analysis of variance (ANOVA). The modeling and optimization of the esterification process were carried out, using RSM of the Design Expert software version 8.0.7.1 (Stat‐ease Inc., MN, USA). Pareto chart was developed using Statistica software, version 12.0 (Stat Soft, Inc., 2014).

#### 2.3.1 Derivation of acetylated starch and process parameters optimization

The acetylation process was carried out as described by Lawal (2004) with some modifications. The extracted native amaranth starch varying from 50 to 150 g (Table 1) was dispersed in 500 ml of distilled water and magnetically stirred for 20 min. The pH of the slurry was adjusted to 8.0. Acetic anhydride varying from 5–15 ml was added while maintaining a pH range of 8.0–8.5. The reaction was allowed to proceed for time varying from 3 to 13 min after the addition of acetic anhydride. Thereafter, the pH of the resultant slurry was adjusted to 7 using 0.1 mol/L HCl solution, centrifuged at 4552 × g, using a centrifuge (0502‐1 Hospibrand) for 15 min, washed with distilled water (1:10 w/v) four times, dried in a hot air oven (Uniscope, SM9053) for 36 hr, milled using attrition mill, sieved with mesh 212 μm and packaged in an airtight plastic container. The flow chart for the process is shown in Figure 1.

### 2.4 Determination of percentage acetylation and degree of substitution

The methods described by Medinav, Pardo, and Ortiz (2012) with some modifications were used. Acetylated starch (1 g) was dispersed in 50 ml of 75% ethanol in a 250‐ml conical flask, stirred in a thermostated water bath (Julabo, model SW22, Germany) at 50°C for 30 min and cooled to 30°C. A 40 ml of 0.5 mol/L KOH and a few drops of phenolphthalein were added. The flask was corked, shaken and allowed to stand for 72 h on more at 30 ± 2°C while it was occasionally swirled. After this, the content was titrated with 0.02 mol/L HCl. The flask was allowed to stand at 30 ± 2°C for 2 hr. Thereafter, the additional alkali which leached from sample was titrated with 0.02 mol/L HCl. The same procedure was carried out with the native starch to serve as a reference. The percentage of acetyl (CH_3_‐C = O^−^) group was calculated as shown in Equation (5) while the degree of substitution (DS) of the acetylated starch was calculated using Equation (6).

(5) $$\%\text{Acetyl}=\frac{\left(\text{Blank}-\text{Sample}\right)\text{ml}\times {\text{mol/L}}_{\mathrm{HCI}}\times 0.043\times 100}{\text{Weight of sample}},$$

(6) $$\text{Degree of substitution}=\frac{\left(162\times \%\text{Acetyl}\right)}{\left(4300-(42\times \%\text{Acetyl})\right)},$$

where, blank is ml of HCl used in the native starch titration, sample is the ml of HCl used in the esterified starch titration, 0.043 is milliequivalents of the acetyl group, 162 is the molecular weight of glucose, 4300 is molecular weight of the acetyl group multiplied by 100, and 42 is the molecular weight of the acetyl group −1.

### 2.5 Modeling and optimization using ANN

A commercial software, NeuralPower version 2.5 (CPC‐X Software), was used for the ANN modeling and optimization. The dependent variables (%acetyl and DS) were predicted by using multilayer full feed forward (MFFF) and multilayer normal feed forward (MNFF) neural networks, which were trained by different learning algorithms such as incremental back propagation (IBP), quickprob (QP), genetic algorithm (GA), batch back propagation (BBP), and Levenberg‐Marquardt algorithm (LM). Each ANN was trained using a default stopping criteria of 100,000 iterations. The dataset in Table 1 was divided into three parts for training (70%), testing (15%), and validating (15%) the network. The inbuilt genetic algorithm in the NeuralPower software was employed in optimization of the independent variables and predictions of the dependent variables. The accuracies of the optimum points predicted were evaluated by comparing values of the predicted‐dependent variables with experimental values, using mean relative percent deviation modulus (*E*), and absolute average deviation (AAD).

(7) $$R^{2}=1-\frac{\sum_{i=1}^{n}{\left(a_{e,i}-a_{p,i}\right)}^{2}}{\sum_{i=1}^{n}{\left(a_{p,i}-a_{e,\mathrm{ave}}\right)}^{2}},$$

(8) $$\text{Adjusted}\;R^{2}=1-\left[\left(1-R^{2}\right)\times \frac{n-1}{n-j-1}\right],$$

(9) $$E\%=\frac{\left[100{\sum_{i=1}^{n}\left(\frac{x_{\mathrm{ei}}-x_{\mathrm{pi}}}{x_{\mathrm{ei}}}\right)}^{2}\right]}{n},$$

(10) $$\text{AAD}\left(\%\right)=\left(\frac{1}{n}\sum_{i=1}^{n}\left(\frac{y_{i,\exp}-y_{i,\mathrm{cal}}}{y_{i,\exp}}\right)\right)\times 100,$$

where, *n* is the number of experimental data, *a*_*p*,*i*_ is the predicted values, *a*_*p,*ave_ is the average predicted values *a*_*e,i*_ is the experimental value, *a*_*e,*ave_ is the average experimental values, and j is the number of input variables (Akanbi, Adeyemi, & Ojo, 2006; Emeko et al., 2015).

## 3 RESULTS AND DISCUSSION

### 3.1 Acetyl content and degree of substitution

The results of the acetyl content and degree of substitution (DS) are presented in Table 1. The acetyl content of the acetylated starch ranged from 0.34 to 2.64%. The results indicated that percentage acetyl increased with increase in the concentration of acetic anhydride in the reaction medium. Similar results were reported by Saartrat, Puttanlek, Rungsardthong, and Uttapap (2005) for kidney bean starch (1.31–4.40%). Wani, Sogi, and Gill (2015) reported a range of 0.80–2.09% for Indian black gram. According to United States Code of Federal Regulations (FAO/JECFA, 2014), the maximum acetyl group allowed in food is 2.50%. The three‐free hydroxyl (OH) groups located at C_2_, C_3,_ and C_6_ have different reactivity. The primary OH attached to C_6_ is more reactive and is acetylated more readily than the secondary ones at C_2_ and C_3_ due to steric hindrance and their affinity for OH groups on the neighboring glucose unit (Miyazaki, Hung, Maeda, & Morita, 2006). According to Xie, Liu, and Cui (2005), acetylation of starch affects its functional properties and starch containing 0.5–2.5% acetyl group usually improves the stability and clarity of sols by increasing the degree of swelling and dispersion of starch granules and also reducing retrogradation.

The DS observed in this work ranged from 0.01 to 0.10. The results were similar to a range of 0.05–0.17 reported by Saartrat et al. (2005) for kidney bean. The difference in DS may be attributed to the difference in amylose content and the intergranular packing of starch (Wani et al., 2015). Like the results obtained for the percentage acetyl group, the DS also increased with increase in the concentration of acetic anhydride in the reaction medium. The DS gives information concerning the number of hydroxyl functions substituted per glucose unit. The level of acetylation is expressed as DS (Singh, Kaur, & McCarthy, 2007). Kalita, Kaushik, and Mahanta (2014) reported that in modified food starches, only very few of the hydroxyl groups are altered and ester groups are attacked at very low DS values.

### 3.2 Modeling and parameters optimization of starch acetylation process by RSM

In order to describe the relationship between the dependent variables (acetyl content and DS) and the independent variables (starch solids, acetic anhydride, and reaction time), the dependent variables were fitted by second‐order polynomial quadratic regression models. By applying multiple regression analysis on the experimental data obtained for the dependent variables (Table 1), the analysis of variance (ANOVA) generated for the models are presented in Table 2. According to Myers and Montgomery (2002) and Fristak, Remenarova, and Lesny (2012), a large F‐value indicates that most of the variations could be explained by the regression equation, whereas a low *p*‐value (*p *< .05) indicates that the model is considered to be statistically significant.

**Table 2:** Regression analysis of acetylated starch

Model terms	% Acetyl			Degree of substitution		
	*F*‐value	*p*‐value	Regression coefficient	*F*‐value	*p*‐value	Regression coefficient
Model	125.27	<.0001^a^	–	25.72	<.0001^a^	–
Constant β_o_	–	–	2.13	–	–	0.084
x_1_	56.32	<.0001^a^	−0.17	17.81	.0018^a^	−7.98 × 10^−3^
x_2_	279.20	<.0001^a^	0.38	48.90	<.0001^a^	0.01
x_3_	13.46	.0043^a^	−0.08	2.37 × 10^−3^	.9621	9.20 × 10^−5^
x_1_x_2_	272.03	<.0001^a^	−0.49	66.93	<.0001^a^	−0.02
x_1_x_3_	10.34	.0093^a^	−0.10	0.38	.5497	1.53 × 10^−3^
x_2_x_3_	105.54	<.0001^a^	−0.30	8.14	.0171^a^	−7.05 × 10^−3^
$x_{1}^{2}$	234.11	<.0001^a^	−0.34	51.94	<.0001^a^	−0.01
$x_{2}^{2}$	189.57	<.0001^a^	−0.30	41.69	<.0001^a^	−0.01
$x_{3}^{2}$	23.20	.0007^a^	−0.11	10.47	.0089^a^	−5.95 × 10^−3^
Lack of fit	1.52	.3296	–	2.91	.1331	–
*R* ^2^	.9912			.9586		
Adj. *R* ^2^	.9833			.9213		
Pred. *R* ^2^	.9549			.7298		
CV	5.14			9.60		
Ad. pred.	39.56			16.39		
Mean	1.62			0.06		
*SD*	0.08			0.01		

x_1_, Starch solid; x_2_, Acetic anhydride; x_3_, Time; *R* ^2^, Coefficient of determination; Adj. *R* ^2^, Adjusted coefficient of determination; Pre. *R* ^2^, Predicted coefficient of determination; CV, Coefficient of variation; Ad. Pre., Adequate prediction.

a Significant factors (*p *< .05).

The fitness and adequacy of the models were judged by the coefficient of *R* ^2^ and significance of lack‐of‐fit. The *R* ^2^ is defined as the ratio of the explained variation to the total variation, a measure of the degree of fit (Chan, Lee, Yap, Wan‐Aida, & Ho, 2009; Wang, Yang, Du, & Yi, 2008). The closer the value of *R* ^2^ to unity, the better the empirical model fits the actual data (Fan, Han, Gu, & Chen, 2007). The adjusted *R* ^2^ is a corrected value for *R* ^2^ after the elimination of the unnecessary model terms. If there are many nonsignificant terms included in the model, the adjusted *R* ^2^′would be remarkably smaller than the *R* ^2^ (Chan et al., 2009; Myers & Montgomery, 2002). Also, the absence of any lack‐of‐fit (*p *> .05) strengthened the reliability of a model.

In the case of acetyl content (Table 2), the model low *p*‐value (<.0001) and F‐value (125.27) indicate the statistical significance of the quadratic model. The *R* ^2^ and adjusted *R* ^2^ were .9912 and .9833, respectively, which illustrate that there were excellent correlations between the independent variables; and the fitted model could describe the independent variables adequately (Chen, Chen, Srinivasakannan, & Peng, 2011; Pishgar‐Komleh, Keyhani, Mostoft‐Sarkari, & Jafari, 2012). The value of *R* ^2^ showed that only 0.88% of the total variation could not be explained by the model. Hence, the quadratic model obtained was adequate to describe the influence of the independent variables on the acetyl content.

The coefficient of variation, CV, which is independent of the unit is defined as the ratio of the standard deviation of estimate to the mean values of the observed response. The CV is a measure of reproducibility and repeatability of the model (Chen, Xiong, Peng, & Chen, 2010; Chen et al., 2011; Pishgar‐Komleh et al., 2012). The CV value of 5.14% observed in this work suggests that the model could be considered reasonably reproducible (CV < 10%) (Chen et al., 2011). The adequate prediction (Ad. Pred.) compares the range of the predicted value at the design points to the average prediction error. Adequate prediction measures signal to noise ratio. A ratio greater than 4 is desirable (Rajmohan & Palanikumar, 2013). The value of the adequate prediction for the acetyl content was significantly greater than 4 (39.56). The lack‐of‐fit test value of 0.3296 was not significant (*p *> .05), which also shows a good fit between experimental data and the model.

In the case of DS, the model *p*‐value of <.0001, *F*‐value (25.72), lack‐of‐fit (0.1331), *R* ^2^ (.9586), Adj. *R* ^2^ (.9213), CV (9.60%) and Ad. Pred. (16.39) confirmed the suitability of the generated model. The *R* ^2^ value indicates that the model could explain up to 95.86% of the total variation in the esterification process. It, therefore, shows that the developed models for the esterification process could adequately define the real behavior of the process and could be used for the prediction of the acetyl content and DS. The mathematical models in coded forms to predict the acetyl content and DS are presented in Equations (11) and (12).

(11) $$\begin{array}{cc} \%\text{Acety}\; & =2.13-0.17x_{1}+0.38x_{2}-0.083x_{3}-0.49x_{1}x_{2}-0.095x_{1}x_{3}\\ & -0.30x_{2}x_{3}-0.34x_{1}^{2}-0.30x_{2}^{2}-0.11x_{3}^{2} \end{array},$$

(12) $$\begin{array}{cc} \text{DS}\; & =0.084-7.975\times 10^{-3}x_{1}+0.013x_{2}+9.201\times 10^{-5}x_{3}\\ & -0.020x_{1}x_{2}+1.529\times 10^{-3}x_{1}x_{3}-7.046\times 10^{-3}x_{2}x_{3}\\ & -0.013x_{1}^{2}-0.012x_{2}^{2}-5.952\times 10^{-3} \end{array}.$$

**Figure 2:**
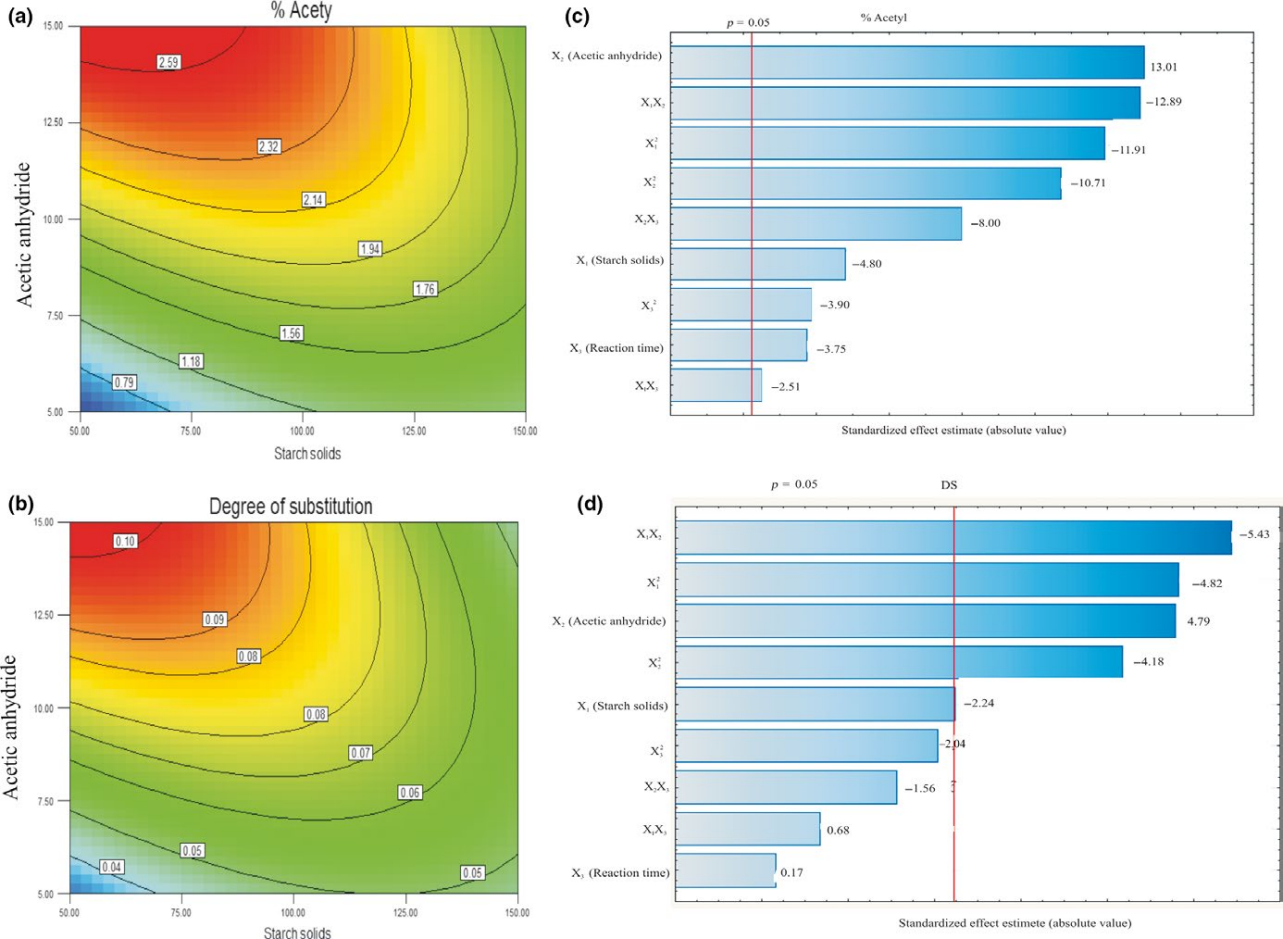
Contour plots and pareto charts for optimization of acetylation of *Amaranthus viridis* starch

Equations (11) and (12) show that starch solids, x_1_; acetic anhydride, x_2_; and reaction time, x_3_ have significant (*p *< .05) influence on the amount of acetyl group present in the starch. Starch solids and reaction time were inversely related to the acetyl content of the esterified starch while the quantity of acetic anhydride was directly proportional. There was significant (*p *< .05) interactions among the independent variables. The DS was significantly (*p *< .05) influenced by amount of starch solids and acetic anhydride. There was positive correlation between acetic anhydride and DS. The DS was also significantly (*p *< .05) influenced by the interactions between starch solids and acetic anhydride; acetic anhydride and reaction time. The quadratic term of all independent variables had significance effect (*p *< .05) on DS. The graphical representations of the regression equations for the optimization of the esterification process are shown in Figure 2a–b. At constant reaction time, for specific mass of starch solids, the formation of starch esters was directly affected by the amount acetic anhydride employed. Similar observations were recorded for DS at a constant reaction time, for a given mass of starch solids.

Pareto charts were developed to evaluate the standardized effects and interactions of starch solids, acetic anhydride and reaction time on acetyl content and DS, and are presented in Figure 2c–d. Figure 2c indicates that all the linear terms, quadratic terms and interaction terms significantly (*p *< .05) influenced the amount of acetyl group in the esterified starch. Acetic anhydride was identified as the most significant process variable on acetyl level of the esterified starch. Figure 2d shows that quadratic model terms of starch solids and acetic anhydride were significant (*p *< .05) and linear model term of acetic anhydride and interaction model term of starch solid x acetic anhydride were significant (*p *< .05). A positive coefficient shows favorable interaction effect on the dependent variable while a negative coefficient shows an inverse relationship between the model term and dependent variable (Emeko et al., 2015).

#### 3.2.1 Optimal values and verification of predictive models

According to United States Code of Federal Regulations (FAO/JECFA, 2014), the maximum acetyl content allowed in foods is 2.5%, and DS <0.1. The regression Equations (11) and (12) were solved using Design Expert Software. The values of the independent variables were estimated and presented in Table 3(a). Starch solids (x_1_) was estimated as 149.57 g, acetic anhydride (x_2_) was 10.83 ml, and reaction time (x_3_) was 3 min; the corresponding acetyl content and DS were 1.61% and 0.06, respectively. The confirmatory experiments conducted under the optimal condition showed acetyl content of 1.68% and DS of 0.06, which were consistent with predicted values. The validity of the predicted optimum points was done using AAD and mean *E*. The AAD values of 4.17% and 0% were recorded for acetyl content and DS, respectively; the values of *E* for acetyl content and DS were 0.17% and 0%, respectively. The small values (<10%) of AAD and *E* for the dependent variables indicated the validity of the model and the developed models are suitable for describing derivation of esterified starch from native starch of *A. viridis*.

**Table 3:** Optimization of acetylation process (a) optimal conditions (b) evaluation of transfer functions

(a)										
	RSM^a^					ANN^b^				
	Actual value	Predicted value	Residual error	AAD^c^ (%)	E^d^ (%)	Actual value	Predicted value	Residual error	AAD (%)	E (%)
Independent										
Starch solids (g), X_1_	–	149.57	–	–	–	–	152.46	–	–	–
Acetic anhydride (ml), X_2_	–	10.83	–	–	–	–	11.00	–	–	–
Reaction time (min), X_3_	–	3.00	–	–	–	–	2.92	–	–	–
Dependent										
Acetyl (%)	1.68 ± 0.01	1.61	0.07	4.17	0.17	1.69	1.74	0.05	2.87	0.09
Degree of substitution	0.06 ± 0.01	0.06	0.00	0.00	0.00	0.06	0.06	0.00	0.00	0.00

(b)								
Model	Learning algorithms	Connection types	Output layer transfer function	Input layer transfer function	Training		Testing	
					e *R* ^2^	fRMSD (%)	*R* ^2^	RMSD (%)
3‐8‐2	IBP^g^	MNFF^h^	Hyperbolic Tangent	Hyperbolic Tangent	.9928	0.01	.9928	0.42
3‐8‐2	IBP	MNFF	Sigmoid	Sigmoid	.9855	0.02	.9719	0.59
3‐8‐2	IBP	MNFF	Linear	Linear	.4570	0.21	.4570	0.98
3‐8‐2	IBP	MNFF	Gaussian	Gaussian	−5.5028	0.05	−11.355	1.66
3‐8‐2	IBP	MNFF	Threshold Linear	Threshold Linear	.8636	0.11	.6298	0.93
3‐8‐2	IBP	MNFF	Bipolar Linear	Bipolar Linear	.4570	0.21	.4574	0.98

a Response surface methodology.

b Artificial neural network.

c Absolute average deviation.

d Mean relative percent deviation modulus.

e Coefficient of determination.

f Root mean square deviation.

g Incremental back propagation.

h Multilayer normal feed forward.

### 3.3 Modeling and parameters optimization of starch acetylation process by ANN

**Figure 3:**
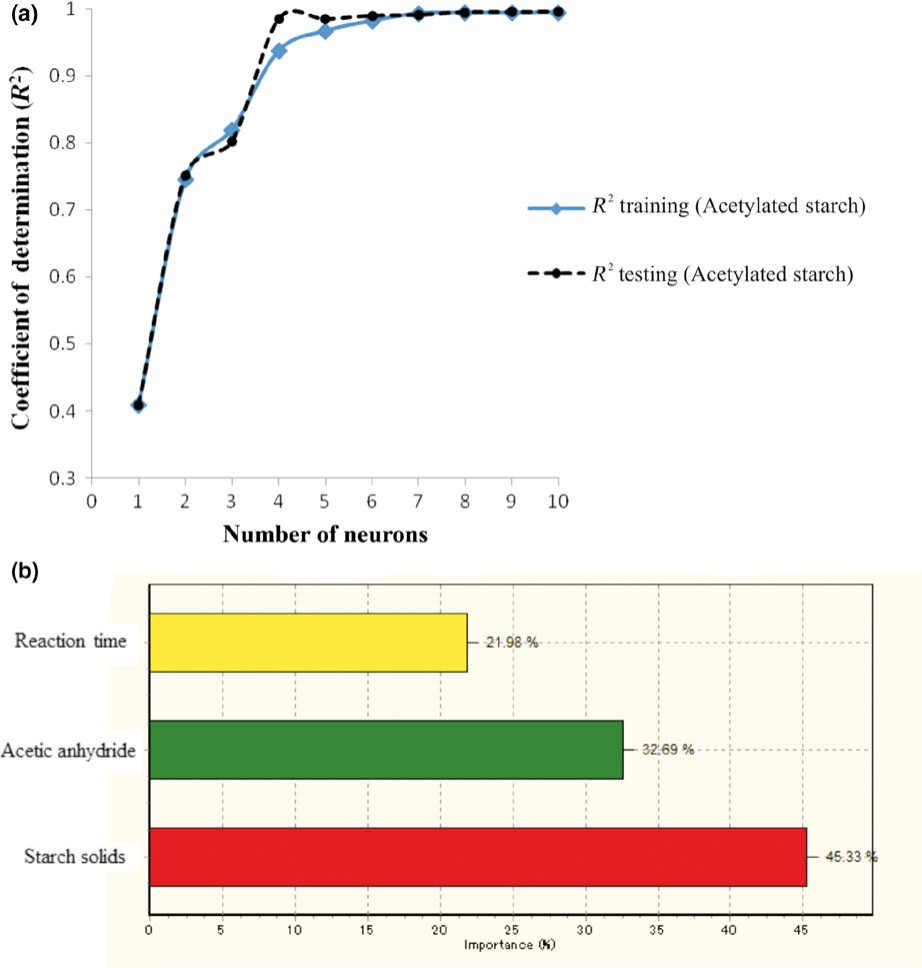
Optimum number of hidden neurons in acetyl content and degree of substitution, topology of multilayer feed forward neural network, and overall evalution of process variables in acetylation of *Amaranthus viridis* starch

In the derivation of esterified starch process, many neural network architectures and topologies for the estimation and prediction of the dependent variables were tested. The ANN models in the optimal region are presented in Table 3(b). As reported by Betiku and Taiwo (2015), there are many learning algorithm types reported in the literature, thus, it is difficult to know in advance which of the learning algorithms will be more efficient for a given study. According to Betiku and Taiwo (2015), the transfer function types employed affect the neural network learning and aid its performance. Thus, several ANN learning algorithms and transfer functions effects were evaluated by successful training of the neural network models. The results obtained indicated that IBP was the most suitable learning algorithm for the dependent variables synthesis (Table 3b). Multiple topologies were examined in order to determine the optimum number of neurons in the hidden layer, in which the neurons were varied from 1 to 10 (Figure 3a). The predictive ability of the network was measured using *R* ^2^ and RMSD. The topology of 3‐8‐2 was identified as the most appropriate, which has eight neurons. The multilayer normal feed forward (MNFF) connection type and incremental back propagation (IBP) network with hyperbolic tangent as both hidden and output layers function was identified as the most suitable ANN model for acetyl content and DS synthesis (Figure 3b).

The values of *R* ^2^ and RMSD for training dataset were 0.9928 and 0.01%, respectively. For the testing dataset, the values were 0.9928 and 0.42%, respectively. This implies that empirical models derived from ANN could be used to describe the input variables for acetyl content and DS synthesis. The predicted conditions were starch solids of 152.56 g, acetic anhydride of 11 ml and reaction time of 2.92 min (Table 3a). The values of 1.74% and 0.06 were predicted for acetyl content and DS, respectively (Table 3a). The predicted values for acetyl content and DS were authenticated by conducting three experimental replicates under the predicted optimal conditions (as it was done in section ACKNOWLEDGMENTS). The average experimental values of 1.69% and 0.06 were obtained for acetyl content and DS, respectively. The prediction was validated by using absolute average deviation (AAD) and mean relative percent deviation modulus (E). The results showed that the low values (value <10%) of AAD and E for the dependent variables indicated that the developed ANN model was effective and adequate for the esterification process. Overall, the most important independent variable for the derivation of esterified starch was acetic anhydride (42.59%), followed by starch solids (33.90%) and then reaction time (23.51%) (Figure 3c).

#### 3.3.1 Performance evaluation of ANN and RSM for the acetylation process

The extent of accuracy of the developed models from RSM and ANN were examined using *R* ^2^ and *E* (Table 3). The average *R* ^2^ of RSM and ANN were .9749 and .9928, respectively; and *E* values of 0.09 and 0.05% for RSM and ANN, respectively. Thus, the ANN model proved to be more effective due to the higher value of *R* ^2^ and lower value of *E*. Similar observations were reported in the modeling and optimization studies on enzymatic reaction catalyzed by amyloglucosidase (Bas & Boyaci, 2007), ethanol production from breadfruit starch (Betiku & Taiwo, 2015) and oxalic acid production from cashew juice (Emeko et al., 2015). Conclusively, ANN performed better than RSM in the modeling and optimization of acetylation process for *A. viridis* starch.

## 4 CONCLUSION

The work examined the acetylation (esterification) of *A. viridis* starch and optimization of the process variables. The acetylation process showed that starch solids, acetic anhydride, and reaction time had significant (*p *< .05) effects on the modification process. The optimal condition of 152.46 g of starch, 11 ml of acetic anhydride, and reaction time of 2.92 min yielded acetyl content of 1.74% and degree of substitution (DS) of 0.06 for the ANN method while optimal condition of 149.57 g (starch), 10.38 ml (acetic anhydride), and 3 min (time) yielded acetyl content of 1.61%, and DS of 0.06 for RSM method. ANN was demonstrated to be a superior modeling tool than RSM. The work provides useful information regarding the development of economic and efficient process for modification of *A. viridis* starch via acetylation method.

## CONFLICTS OF INTEREST

The authors declare no conflicts of interest.

## ETHICAL STATEMENT

Not applicable.
